# Characteristics and outcome in patients with non-specific symptoms and signs of cancer referred to a fast track cancer patient pathway; a retrospective cohort study

**DOI:** 10.1186/s12885-017-3826-z

**Published:** 2017-12-02

**Authors:** Sara Falk Jørgensen, Pernille Ravn, Søren Thorsen, Signe Westring Worm

**Affiliations:** 1 Department of Pulmonary and Infectious Diseases, University Hospital, North Zealand Hospital, Hillerød, Denmark; 2 Faculty of Health and Medical Sciences, Copenhagen University, Copenhagen, Denmark; 3 Department of Infectious Diseases, University Hospital Rigshospitalet, Copenhagen, Denmark

**Keywords:** Cancer, Fast-track, Non-specific symptoms, Denmark, One-year mortality

## Abstract

**Background:**

In 2012 a new cancer patient pathway for patients with non-specific symptoms and signs of cancer (NSSC-CPP) was introduced in Denmark. Limited information is available about the patients referred to the NSSC-CPP and the investigational course. The aim was to describe the population and the investigational course, estimate the prevalence of cancer and one-year mortality, and identify factors associated with a subsequent cancer diagnosis in patients referred to the NSSC-CPP.

**Method:**

This cohort study included patients with at least one visit at the NSSC-CPP at North Zealand Hospital in Denmark (NOH) from October 1st 2013 to September 30th 2014. Data was based on retrospective reviews of the patient files. Logistic regression identified factors associated with a subsequent cancer diagnosis. Multivariate analyses were adjusted by age, gender, smoking status and alcohol consumption. Kaplan-Meier survival plots were made at one-year follow-up.

**Results:**

Eight hundred twenty-five patients were included with a median age of 67 years, 47.4% were male. Prevalence of cancer within one year was 16.7% (138/825). 70.3% (97/138) were solid cancers and 29.7% (41/138) were haematological cancers. During the investigational course 76.7% went through advanced diagnostic imaging (ultrasound, CT, FDG-PET/CT or MRI). Anaemia (OR1.63 CI1.02–2.60), leucocytosis (OR 2.06 CI 1.34–3.15), thrombocytopenia (OR 4.13 CI 2.02–8.47) and elevated LDH (OR 1.64 CI 1.07–2.52) and CRP (OR 2.56 CI 1.66–3.95) were associated with a cancer diagnosis when adjusting for possible confounders. No single non-specific symptom was significantly associated with a cancer diagnosis. One-year mortality for those diagnosed with cancer was 44.2%.

**Conclusion:**

The prevalence of cancer matches that of another NSSC-CPP in Denmark. Deviations in basic biochemistry were associated with a higher probability of underlying cancer and could possibly raise the level of suspicion of malignancy among physicians. High one-year mortality was seen amongst patients diagnosed with cancer.

## Background

Fast track investigational courses for patients with suspected cancer have been implemented in several European countries [1–3]. In United Kingdom (UK) the 2-week wait (2WW) referral systems was introduced in 2000, and in Denmark organ-specific Cancer Patient Pathways (CPP’s) were implemented in 2007 [2, 3]. Despite these efforts British and Danish cancer patients suffer from low cancer survival rates in comparison to other western countries [4–8]. Not all cancer patients have benefitted from the implementation of organ-specific CPPs [6, 7, 9, 10], and a high proportion of malignancies have previously been diagnosed outside the CPPs [6, 11, 12]. One in every fourth cancer patient present with non-organ specific symptoms (e.g. pain, weight loss or fatigue) causing the general practitioner to suspect a serious disease [11]. These patients are not eligible for referral to organ-specific CPP’s.

Patients presenting with non-specific symptoms have a longer time to diagnosis and lower survival rates compared to patients presenting with organ-specific symptoms [13]. Therefore a new CPP for patients with non-specific symptoms and signs of cancer (NSSC-CPP) was implemented in Denmark in 2012 [12, 14, 15]. The goal of the NSSC-CPP was to ensure an accelerated investigational course of no longer than 22 days, for patients presenting with non-specific symptoms and signs of cancer [14].

Organ-specific symptoms, such as bleeding from the intestinal tract and persisting digestive problems have low predictive values of cancer [16–19], and some patients will experience warning symptoms without an underlying cancer [20]. Whether non-specific symptoms and other patient characteristics are related to a cancer diagnosis in the NSSC-CPP setting, is yet unknown. New tools are needed in the diagnostic process to determine which patients are at highest risk of having cancer.

No formal guidelines for the investigational course at the NSSC-CPP have yet been made. As of now the diagnostic course includes blood tests and imaging as found relevant by the physician in charge. The use of Computed Tomography (CT) and Positron Emission Tomography, with different tracers, in combination with CT (PET/CT) have proven valuable in studies regarding fever of unknown origin (FUO) and in the diagnostic process and staging of several solid cancers [21–28]. The use of imaging in the NSSC-CPP setting has not yet been determined. Basic biomarkers such as haemoglobin, leukocytes, thrombocytes, CRP and LDH have proven to have prognostic value in many cancers, whereas their predictive values have not yet been examined in the NSSC-CPP setting [29–35].

Research in the NSSC-CPP setting has previously focused on the general practitioners (GP’s) part of the diagnostic process or on a limited number of patients. These studies show that the GP’s gut feeling was a valuable indicator of the likelihood of cancer, and found cancer rates of 16–18% [36–38]. Finally the survival-rate in patients seen at the NSSC-CPP has not yet been determined.

The aim of this study was to describe the population referred to the NSSC-CPP and the investigational course, estimate the prevalence of cancer and one-year mortality and identify factors associated with a subsequent cancer diagnosis in these patients with non-specific symptoms and signs of cancer.

## Methods

The study was a single centre cohort study on patients referred to the NSSC-CPP at a university hospital, North Zealand Hospital (NOH), in the capital region of Denmark. Study period covered from October 1st 2013 to September 30th 2014. Patient files were re-evaluated after one year; files of patients with a cancer diagnosis were re-evaluated one year after the time of diagnosis.

### The NSSC-CPP setting in the capital region of Denmark

The population of Denmark is entitled to public health-care benefits including free access to health-care. The outpatient-clinic handling the NSSC-CPP at the University Hospital, North Zealand Hospital (NOH) has a catchment area of 310.000 citizens covering 19% of the capital region of Denmark. Patients with non-organ-specific symptoms and signs of cancer, who were healthy enough for an outpatient course, were referred to the NSSC-CPP by their GP and, or by other hospital departments. A predefined set of blood samples and a chest x-ray was required before the first visit. On basis of the information available at referral the physician at the NSSC-CPP decided whether additional testing, including imaging should be made before the patients attended their first visit. During first consultation further investigations were planned. A coordinating nurse and secretary made all appointments and arrangements, and all patients were interviewed and examined by a subgroup of specialists at the Department of Pulmonary and Infectious Diseases, dedicated to the NSSC-CPP.

After a finalized investigational course the patient was categorized into one of four groups i) cancer no longer suspected (ICD10 codes (International Classification of Diseases 10th Revision) DZ031 and ZZ5650), ii) cancer was diagnosed and the patient was referred for treatment or further diagnostic efforts at an organ-specific CPP, iii) Patient was still strongly suspected of having cancer and was referred to an organ-specific CPP (ICD10 code DZ031XX), iv) Patient was still suspected of having cancer, but not found suitable for a fast track investigational course, or the patient did not want further investigation at all.

### Inclusion and exclusion

During the study period a list with the unique identification number of every patient referred to the NSSC-CPP was created. Among those referred to NSSC-CPP, electronic patient files were checked to identify patients above 18 years of age, with no new biopsy verified cancer at referral and with at least one visit at the NSSC-CPP. Patients with a previous cancer diagnosis were assessed both by the GP and the physician receiving the referral and if their symptoms were not obviously related to their prior cancer and they were found eligible by the above mentioned criteria they were included in the study. Patients were only included once.

### Data collection

Data were collected retrospectively by review of the patient files (both paper forms and electronic files). Relevant information of the course of investigation was collected; i.e. symptoms, clinical findings, laboratory results, use of imaging, findings by imaging, pathologic examinations, endoscopies, concluding diagnoses and status at one-year follow-up. The final diagnosis for those patients without cancer diagnosis was defined as the diagnosis found most likely to explain the patient’s symptoms. The decision made by the investigating physician at NSSC-CPP, or by the department taking over the investigational course after the NSSC-CPP. Cancer diagnoses entered in the database were any cancer diagnosis given within one year after ended investigational course at the NSSC-CPP.

All diagnoses were crosschecked: The paper forms filled out by the investigating physician was compared to the electronic patient files and the Patient Index (where the patients ICD-10 codes were listed). A standard operating procedure (SOP) was made. In order to ensure standardization of the gathering and entering of data, all complicated cases were gathered and discussed amongst the study group and conclusions were entered in the SOP. Information not available in the form filled out by the investigating physician or in the electronic patient files was recorded as missing.

Data were entered into a database using Epidata (www.epidata.dk).

National guidelines on alcohol intake were used as cut off value in terms of alcohol consumption [39]. ICD-10 codes DC00-DC97 were regarded as cancer diagnoses. Concluding diagnoses and diagnoses at follow-up were crosschecked in terms of correlation between the paper files, the electronic patient files and the Patient Index (where the patients ICD-10 codes were listed). Information not available in the patient files was noted as missing.

### Statistics

Chi-square (X^2^)/Fishers exact test and Wilcoxon rank-sum test were used to identify differences in the distribution of characteristics between patients with and without a cancer diagnosis. Data were presented as percentages (counts) and means/medians (95% Confidence Interval (CI)/inter quartile range (IQR)). Cancer probability was presented as the percentage of included patients with a cancer diagnosis or relapse of a previously diagnosed cancer within one year from ended investigational course at the NSSC-CPP. Patients given the concluding diagnostic codes DZ031 and ZZ5650 – cancer is no longer suspected, by the NSSC-CPP, but who were subsequently diagnosed with cancer (within one year), were regarded as cancers not detected by the NSSC-CPP.

Logistic regression was used to find associations between cancer diagnosis and patient characteristics, symptoms and basic biochemistry abnormalities. Multivariate analyses were adjusted by age, gender, smoking status and alcohol consumption - covariates proven to have impact on cancer risk in previous literature [40, 41]. Sensitivity analyses were additionally adjusted by the variable ‘previously diagnosed cancer’. Additional sensitivity analyses examined the association of characteristics, symptoms and basic biochemistry abnormalities with solid and haematological cancer diagnoses respectively. For haematological cancer, patients with solid cancer and patients with no cancer diagnosis were used as combined reference group. For solid cancer patients with haematological cancer and patients with no cancer diagnosis were used as a combined reference group. Statistical significance level was set at a *P*-value of <0.05.

Kaplan Meier curves were made to estimate one-year survival and mortality in patients with a cancer diagnosis and patients with no cancer diagnosis. Follow-up time for patients with no subsequent cancer diagnosis started at the conclusion of the diagnostic work-up. For patients with at subsequent cancer diagnosis, follow-up started at time of diagnosis.

SAS Enterprise Guide 7.1 was used for the statistical analyses.

### Ethics and approvals

This study was approved by the Danish Data Protection Agency (j.nr. 2012–58-0004). Written informed consent was not obtained from the human subjects do to the retrospective design. Approval to go through patient files were instead given by the Danish Health and Medicines Authority (j.nr. 3–3013-1195/1/). Approval from the Danish National Committee on Health Research Ethics was, according to national guidelines, not needed as no biomedical intervention was performed.

## Results

### Study population

**Fig. 1:**
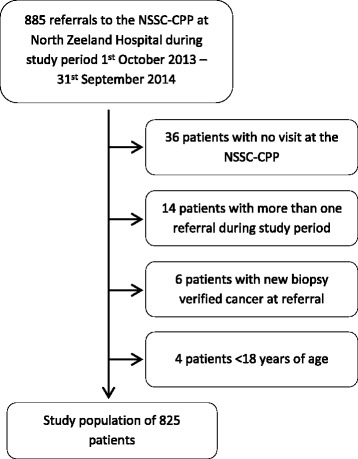
Inclusions and exclusions. ^1^NSSC-CPP= cancer patient pathway for patients with nonspecific symptoms and signs of cancer

Eight hundred eighty-five patients were referred to the NSSC-CPP at NOH during the study period and 825(93%) were included in the study (Fig. 1).

### Patient characteristics

The median age was 67 (IQR 55–75) years and 47.4% were male. The population was primarily referred to the NSSC-CPP from a GP (75.4%). Current or former smoking was reported in 65.8% and 9.5% had a weekly alcohol consumption level above national guidelines. Cardiovascular disease (15.6%), lung disease (13.3%) and previously diagnosed cancer (12.2%) were the most common comorbidities.

One year after ended diagnostic course at the NSSC-CPP 16.7% (138) of the patients had been diagnosed with cancer. Patients diagnosed with cancer were significantly older and more often previously diagnosed with cancer (Table 1).

**Table 1:** Characteristic in patients with and without cancer, *P*-value representing a test for difference

Variable	All	Cancer^a^	No-cancer^a^	*P*-value
% (N)	100 (825)	16.7 (138)	83.3 (687)	
Referred by				
General practice	75.4 (605)	74.6 (97)	75.6 (508)	0.851
Hospital department	20.1 (161)	21.5 (28)	19.8 (133)	
Other	4.5 (36)	3.8 (5)	4.6 (31)	
Gender				
Male	47.4 (391)	44.2 (61)	48.0 (330)	0.411
Age				
median (IQR)	67 (55–75)	69 (62–76)	67 (53–74)	0.003
Groups				
18–39 years	7.5 (62)	3.6 (5)	8.3 (57)	0.050
40–54 years	17.1 (141)	10.9 (15)	18.3 (126)	
55–69 years	33.0 (272)	36.2 (50)	32.3 (222)	
70–79 years	31.0 (256)	36.2 (50)	30.0 (206)	
≥ 80 years	11.4 (94)	13.0 (18)	11.1 (76)	
Body mass index				
median (IQR)	24.3 (21.7–28.0)	23.4 (21.9–26.8)	24.5 (21.7–28.1)	0.317
Smoking status				
Never	34.2 (271)	29.6 (40)	35.1 (231)	0.222
Former/current	65.8 (522)	70.4 (95)	64.9 (427)	
Package years median (IQR)^b^	25 (15–40)	30 (14–40)	25 (15–40)	0.818
Alcohol consumption per week				
Units per week, median (IQR)^c^	5 (0–14)	7 (0–14)	4 (0–14)	0.378
Alcohol consumption above recommendations^d^	9.5(69)	11.3 (13)	9.1 (56)	0.466
Chronic diseases at first visit				
Lung disease	13.3 (110)	10.1 (14)	14.0 (96)	0.227
Cardiovascular diseases	15.6 (129)	11.6 (16)	16.4 (113)	0.152
Cerebrovascular diseases	10.8 (89)	12.3 (17)	10.5 (72)	0.525
Diabetes	11.6 (96)	8.7 (12)	12.2 (84)	0.238
Inflammatory diseases	11.9 (98)	7.2 (10)	12.8 (88)	0.065
Renal failure	4.1 (34)	2.2 (3)	4.5 (31)	0.207
Peptic ulcer	3.2 (26)	3.6 (5)	3.1 (21)	0.788
Cirrhosis	0.4 (3)	0.7 (1)	0.3 (2)	0.423
Dementia	1.8 (15)	0	2.2 (15)	0.152
Previously diagnosed cancer	12.2 (101)	21.7 (30)	10.3 (71)	<0.001
Number of chronic diseases				
0	46.3 (382)	44.9 (62)	46.6 (320)	0.054
1–2	46.3(382)	52.2 (72)	45.1 (310)	
≥ 3	7.4 (61)	2.9 (4)	8.3 (57)	

Data presented as percentages (counts) unless otherwise indicated. Chi square-test or Fishers exact test for 2 × 2 tables, Students T-test for normally distributed data, otherwise Wilcoxon’s Rank Sum Test. ^a^Cancer or no-cancer within one year after ended investigational course. ^b^In current or former smokers only. ^c^One unit = 15 ml of alcohol. ^d^Recommendations = national guidelines. Less than 5% missing values in every variable except for Body Mass Index (32.5%), Package years (12.8%) and alcohol consumption (11.8%)

Weight loss (cancer group 39%, no-cancer group 42%), fatigue (cancer group 35% no-cancer group 39%) and loss of appetite (cancer group 28%, no-cancer group 26%) were the most common symptoms in both groups. No single symptom was significantly more or less pronounced in the cancer group (data not shown).

Objective findings were inconsistently reported or performed (Rectal exploration: 22%, breast examination: 60% of all women, auscultation: 82%, abdominal examination: 78%, lymph nodes: 82%, data not shown).

### The investigational course

The duration of the diagnostic course at the NSSC-CPP was a median of 9 days (IQR 1–15), and patients had a median of 2 visits (IQR 1–2). During the investigational course 76.7% of the patients went through advanced imaging (CT, FDG-PET/CT, Ultrasound or Magnetic Resonance Imaging (MRI)). FDG-PET/CTs and CTs were the preferred type of imaging, used in 30.4% and 39.0% of the investigational courses respectively. FDG-PET/CTs more often in patients subsequently diagnosed with cancer (Table 2).

**Table 2:** Investigational course, *P*-value representing a test for difference

	All	Cancer^a^	No-cancer^a^	*P*-value
	*N* = 825	*N* = 138	*N* = 687	
Length of investigational course in days median (IQR)^b^	9 (1–15)	10 (1–16)	9 (1–15)	0.699
Number of visits at the NSSC-CPP median (IQR)	2 (1–2)	2 (1–2)	2 (1–2)	0.960
Diagnostic imaging used during or prior to the investigational course %(n)				
CT	39.0 (322)	42.8 (59)	38.3 (263)	0.326
FDG-PET/CT	30.4 (251)	38.4 (53)	28.8 (198)	0.026
Ultrasound	16.0 (132)	10.9 (15)	17.0 (117)	0.072
MRI	5.3 (44)	2.9 (4)	5.8 (40)	0.163
No advanced imaging	23.3 (192)	22.5 (31)	23.4 (161)	0.805
Pathological examinations and endoscopies performed during investigational course %(n)				
Bone marrow	13.3 (110)	21.7 (30)	11.6 (80)	0.002
Lymph node extirpation	2.8 (23)	9.4 (13)	1.5 (10)	<0.001
Lower endoscopy^c^	9.9 (82)	8.0 (11)	10.3 (71)	0.397
Upper endoscopy^c^	9.6 (79)	5.0 (7)	10.5 (72)	0.049

^a^Cancer or no-cancer within one year after ended diagnostic examination, ^b^From date of first visit, ^c^With or without biopsy. There are less than 5% missing values in every variable except for LDH (5.6%) and Erythrocyte Sedimentation Ratio (48.8%)

The abnormal biochemistry levels seen most often were elevated ESR in 52.4% followed by elevated LDH in 32.8% and elevated CRP in 30.5%. Few patients had leukocytopenia (2.6%) and thrombocytopenia (5.9%). Abnormalities in basic biochemistry were seen more often in the cancer group.

Bone marrow examinations were performed in 13.3% of the investigational courses, most often in patients with a subsequent cancer diagnosis (Table 2).

### Outcome and mortality

Overall 16.7% (138) were diagnosed with cancer within one year from finalized diagnostic course at the NSSC-CPP, of those 8% represented a relapse of a previously diagnosed cancer. Time to registration of a cancer diagnosis was a median of 22 days (IQR 10–45) from date of first visit. Among the 138 patients who were diagnosed with cancer 70.3% had a solid cancer and 29.7% had haematological cancer. Gastro intestinal cancer (23.2%) and lung cancer (10.1%) were the most frequent solid cancers. In patients with no subsequent cancer diagnosis the most frequent diagnoses were rheumatic (12.4%), gastrointestinal (10.3%), haematological (8.7%) and infectious (3.5%).

In 9 patients the cancer diagnosis was not detected by the NSSC-CPP. Diagnoses in those 9 patients were as following; bile duct cancer, hepatocellular cancer, basocellular skin cancer, relapse of prostate cancer, ovarian cancer with metastasis, rectal cancer, salivary duct cancer and breast cancer.

**Fig. 2:**
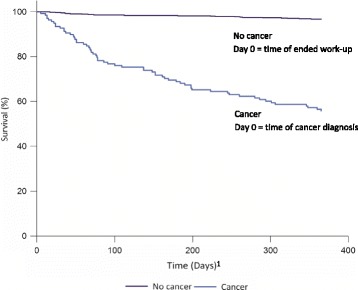
Survival at one-year follow-up. ^1^Risk time: one year from ended work-up at the NSSC-CPP for patients with no subsequent cancer but one year from time of diagnosis for patients with a subsequent cancer diagnosis. NSSC-CPP = cancer patient pathway for patients with non-specific symptoms and signs of cancer

One-year mortality in patients diagnosed with cancer or relapse of cancer was 44.2% and 3.3% for those with no cancer diagnosis (Fig. 2). A sensitivity analysis omitting patients with a previously diagnosed cancer did not change the one-year mortality (data not shown).

### Predictors of cancer

#### Univariate

Age was significantly associated with a cancer diagnosis (OR 1.03, 95%CI 1.01–1.04), with a 34% increase in odds with every ten-year increase in age. No other patient characteristic or single symptom was significantly associated with a cancer diagnosis. Anemia (OR 1.56, 95%CI 1.05–2.31), leukocytopenia (OR 3.45, 95%CI 1.15–10.39), leukocytosis (OR 2.38, 95%CI 1.62–3.50), thrombocytopenia (OR 3.47, 95%CI 1.77–6.81), thrombocytosis (OR 1.89, 95%CI 1.16–3.07) and elevated ESR (OR 1.82, 95%CI1.05–3.15), CRP (OR 2.70, 95%CI 1.84–3.97) and LDH (OR 1.90, 95%CI 1.30–2.79) were significantly associated with a cancer diagnosis in a univariate analysis (Table 3).

**Table 3:** Predictors of cancer diagnosis within one-year^a^

		Unadjusted analysis		Adjusted analysis^b^	
		OR (95%CI)	*P*-value	OR (95% CI)	*P*-value
Age in years		1.03 (1.01–1.04)	<0.001	1.03 (1.01–1.04)	0.002
Gender male		0.86 (0.59–1.24)	0.414	–	–
Alcohol consumption above guidance		1.27 (0.67–2.40)	0.467	–	–
Former/current smoker yes		1.29 (0.86–1.92)	0.223	–	–
Symptoms					
Weight loss		0.89 (0.61–1.31)	0.549	–	–
Fatigue		0.83 (0.56–1.23)	0.348	–	–
Loss of appetite		1.11 (0.73–1.68)	0.626	–	–
Abdominal pain		1.39 (0.88–2.18)	0.158	–	–
Indefinable pain		1.18 (0.75–1.87)	0.479	–	–
Night sweats		0.63 (0.37–1.08)	0.092	–	–
General illness		0.86 (0.50–1.45)	0.560	–	–
Fever		0.50 (0.21–1.18)	0.114	–	–
Other		1.24 (0.72–2.16)	0.440	–	–
Abnormal biochemistry levels					
Anemia	<7.3 for women <8.3 for men	1.56 (1.05–2.31)	0.028	1.63 (1.02–2.60)	0.040
Leucocytopenia	<3.5 × 10^9^	3.45 (1.15–10.39)	0.028	2.01 (0.52–7.74)	0.311
Leucocytosis	>8.8 × 10^9^	2.38 (1.62–3.50)	<0.001	2.06 (1.34–3.15)	<0.001
Thrombocytopenia	<145 × 10^9^	3.47 (1.77–6.81)	<0.001	4.13 (2.02–8.47)	<0.001
Thrombocytosis	>390 × 10^9^	1.89 (1.16–3.07)	0.010	1.67 (0.96–2.91)	0.071
Elevated ESR^c^	>15	1.82 (1.05–3.15)	0.033	1.29 (0.70–2.37)	0.411
Elevated LDH^d^	> 205 U/l	1.90 (1.30–2.79)	0.001	1.64 (1.07–2.52)	0.023
Elevated CRP^e^	>10 mg/l	2.70 (1.84–3.97)	<0.001	2.56 (1.66–3.95)	<0.001

^a^Within one year of ended investigational course at the NSSC-CPP ^b^Multivariate analysis adjusted for age, gender, smoking status and alcohol consumption. ^c^ *ESR* Erythrocyte Sedimentation Ratio. ^d^ *LDH* Lactate dehydrogenase. ^e^ *CRP* C-reactive protein

#### Multivariate

When adjusting for age, gender, smoking status and alcohol consumption, anemia (OR 1.63, 95%CI 1.02–2-60), leukocytosis (OR 2.06, 95%CI 1.34–3.15), thrombocytopenia (OR 4.13, 95%CI 2.02–8.47), elevated LDH (OR 1.64, 95%CI 1.07–2.52) and CRP (OR 2.56, 95%CI 1.66–3.95) were still significantly associated with a cancer diagnosis. Age continued to be associated with a cancer diagnosis when adjusting for gender, smoking status and alcohol consumption (Table 3). Sensitivity analyses additionally adjusting for previously diagnosed cancer did not change the results of the multivariate analyses (data not shown).

In a sensitivity analysis anemia (OR 2.36, 95%CI 1.09–5.08), leukocytopenia (OR 6.98, 95%CI 1.69–28.69) and thrombocytopenia (OR 7.80, 95%CI 3.19–19.10) were significantly associated with a haematological cancer diagnosis when adjusting for possible confounders. Leukocytosis (OR 2.19, 95%CI 1.35–3.55), Thrombocytosis (OR 1.93, 95%CI 1.06–3.51) and CRP (OR 2.91, 95%CI 1.76–4.80) were associated with a solid cancer diagnosis (Table 4).

**Table 4:** Sensitivity analysis - Predictors of solid and haematological cancer within one-year^a^

	Haematological cancer				Solid cancer			
	Unadjusted analysis		Adjusted analysis^b^		Unadjusted analysis		Adjusted analysis^b^	
	OR (95%CI)	*P*-value	OR (95%CI)	*P*-value	OR (95%CI)	*P*-value	OR (95%CI)	*P*-value
Age in years	1.02 (0.99–1.05)	0.069	–	–	1.02 (1.01–1.04)	0.006	1.02 (1.01–1.04)	0.014
Gender male	1.30 (0.69–2.44)	0.411	–	–	0.72 (0.47–1.11)	0.133	–	–
Alcohol consumption above guidance	0.92 (0.27–3.09)	0.894	–	–	1.40 (0.69–2.87)	0.351	–	–
Former/current smoker yes	0.92 (0.47–1.81)	0.816	–	–	1.46 (0.91–2.35)	0.120	–	–
Symptoms								–
Weight loss	0.56 (0.27–1.14)	0.109	–	–	1.09 (0.70–1.69)	0.698	–	–
Fatigue	0.80 (0.41–1.58)	0.522	–	–	0.86 (0.55–1.35)	0.511	–	–
Loss of appetite	0.31 (0.11–0.88)	0.028	0.17 (0.04–0.71)	0.015	1.63 (1.03–2.57)	0.036	1.52 (0.93–2.50)	0.097
Abdominal pain	0.36 (0.11–1.19)	0.094	–	–	1.99 (1.22–3.25)	0.006	2.39 (1.42–4.06)	0.001
Indefinable pain	1.50 (0.72–3.16)	0.281	–	–	1.03 (0.59–1.78)	0.926	–	–
Night sweats	0.34 (0.10–1.13)	0.079	–	–	0.81 (0.45–1.44)	0.465	–	–
General illness	0.43 (0.13–1.40)	0.159	–	–	1.09 (0.61–1.94)	0.769	–	–
Fever	0.61 (0.14–2.58)	0.499	–	–	0.48 (0.17–1.36)	0.169	–	–
Other	0.86 (0.29–2.46)	0.771	–	–	1.41 (0.76–2.59	0.276	–	–
Basic Biochemistry^c^								
Anemia	2.72 (1.43–5.18)	0.002	2.36 (1.09–5.08)	0.029	1.11 (0.69–1.78)	0.669	–	–
Leukopenia	11.69 (3.69–37.00)	<0.001	6.98 (1.69–28.69)	0.007	–^d^	0.987	–	–
Leukocytosis	1.26 (0.62–2.59)	0.521	–	–	2.76 (1.79–4.26)	<0.001	2.19 (1.35–3.55)	0.001
Thrombocytopenia	6.52 (2.83–15.01)	<0.001	7.80 (3.19–19.10)	<0.001	1.53 (0.62–3.76)	0.359	–	–
Thrombocytosis	0.85 (0.29–2.48)	0.767	–	–	2.27 (1.35–3.84)	0.002	1.93 (1.06–3.51)	0.032
Elevated ESR^e^	1.26 (0.49–3.21)	0.622	–	–	2.02 (1.06–3.87)	0.033	1.35 (0.66–2.75)	0.406
Elevated LDH^f^	1.64 (0.86–3.15)	0.137	–	–	1.88 (1.21–2.93)	0.005	1.51 (0.92–2.48)	0.100
Elevated CRP^g^	1.63 (0.84–3.14)	0.147	–	–	3.00 (1.93–4.67)	<0.001	2.91 (1.76–4.80)	<0.001

^a^Within one year of ended investigational course at the NSSC-CPP ^b^Multivariate analysis adjusted for age, gender, smoking status and alcohol consumption. ^c^References are given in Table 3. ^d^Leukopenia were so rarely seen in patients with solid cancer making it impossible to estimate OR. ^e^ *ESR* Erythrocyte Sedimentation Ratio. ^f^ *LDH* Lactate dehydrogenase. ^g^ *CRP* C-reactive protein

## Discussion

### Main findings

Eight hundred twenty-five patients were seen at the NSSC-CPP during the study period with a cancer prevalence of 16.7%. Solid cancers were seen in 70.3%; gastro intestinal and lung cancer being the most common types. Abnormal basic biochemistry levels including anemia, leucocytosis, thrombocytopenia and elevated LDH and CRP were significantly associated with a cancer diagnosis when adjusting for possible confounders. In a sensitivity analysis we found cytopenia (anemia, leukopenia and thrombocytopenia) to be significantly associated with haematological cancer, leucocytosis, thrombocytosis and elevated CRP were associated with solid cancer. Patients diagnosed with cancer had a one-year mortality of 44.2%.

### Patient characteristics

Characteristics of the two groups – cancer and no cancer were surprisingly identical in terms of gender, smoking status and alcohol consumption. Patients referred to the NSSC-CPP were equally ill in terms of comorbidities and symptoms, with the exception of previously diagnosed cancer. Similar findings have been reported by Ingeman et al. who also found weight loss, fatigue and loss of appetite to be the most common symptoms [36], which obviously relates to the fact, that the NSSC-CPP was designed for patients with these symptoms. The equality in comorbidity burden and in symptom presentation might reflect that both groups represented complicated cases where the GP had had trouble finding the right time and place for referral.

### Investigational course

PET/CT (using different tracers) and CT have been recognised as useful tools in diagnosing and staging of many solid cancers and in the FUO-setting [21–28] and FDG-PET/CT’s and CT’s were also the most common choice of imaging in this study. In 23.3% of the patients no advanced imaging was made. This may partly be explained by bone marrow examination being the examination of choice in patients with suspected haematological illness. Determining the usefulness of systematic use of imaging in the setting of the NSSC-CPP in a prospective study is needed.

The length of the investigational course from first visit were a median of 9 days (IQR 1–15). Diagnostic imaging was usually performed before attending first visit at the NSSC-CPP, and the subsequent assessment required a median of merely 2 visits with a specialist, indicating that this type of fast track evaluation is possible. In addition, a substantial effort was made before and between visits by the coordinating nurse and the physician at interdisciplinary conferences and through evaluation of interim test results.

Clinical findings were inconsistently reported. To learn more about the diagnostic yield of different investigations, there is a need for prospective and systematic assessment of these patients.

### Cancer prevalence and mortality

A cancer prevalence of 16.7% is similar to other studies previously examining patients referred to or seen by the NSSC-CPP and finding a cancer prevalence of 16 to 18%, these two studies from the same region of Denmark partly included the same patients [36, 37]. One could argue that this percentage is low, compared to the organ specific CPP’s, with cancer prevalences of 27–30% [42]. An increasing proportion of patients continues to be referred to the NSSC-CPP, it is likely that the cancer prevalence will be reduced slightly. In previous studies as well as ours lung, gastrointestinal and haematological cancers were the most common cancer diagnoses, we however found a higher prevalence of haematological cancer [36, 37]. This could indicate that a high level of suspicion was required in the NOH setting for patients to access the haematological CPP. The most common non-malignant diagnoses were rheumatic, gastrointestinal, non-malignant haematological or infectious (in that order). This matches to some extend findings from a previous study [37].

The one-year mortality of 44.2% in patients with a cancer diagnosis is high considering the short investigational course with no unreasonable delays and is not in line with the aim of finding the cancer diagnoses at curable stages [14, 43]. In comparison the overall one-year mortality for all cancer types between 2009 and 2013 were 23% [44]. Experiences from the organ-specific CPP’s and the UK 2WW-referral system have shown that cancers were not convincingly found at earlier stages after the implementation of these pathways [4–8, 43]. This might also be the case with the NSSC-CPP. A previous study found that patients with non-specific symptoms had a long course leading up to the referral to the NSSC-CPP [36]. Evidently both patient and doctors delays may adversely affect the potential effect of the NSSC-CPP on cancer survival.

### Predictors of cancer

Age was found to have a strong association with a cancer diagnosis which is well known [36, 37, 40].

No single non-specific symptom was significantly associated with a cancer diagnosis. Even organ-specific symptoms are known to have low predictive values of cancer making it unlikely for non-specific symptoms to be highly predictive. Non-specific symptoms are seen very often by the GP and most often in patients with no underlying cancer and the threshold for referring patients is still unknown [11, 16–19]. More experience and knowledge about events prior to referral may provide us with better tools to differentiate who to refer and who not to refer.

Our results suggest that deviations in basic biochemistry levels could be useful predictors of cancer. In line with this Bislev et al. found anemia and elevated alkaline phosphatases associated with a cancer diagnosis in the NSSC-CPP [37]. Basic biochemistry levels are prognostic (and not diagnostic) factors in many specific cancers and might be indicative of advanced stages of cancer and higher risk of deadly outcome [29–35]. In this cohort many cancer patients were seen with abnormal levels in basic biochemistry and a high mortality indicating that these patients despite efforts are diagnosed in advanced stages of their disease. Abnormal levels in basic biochemistry should raise awareness by the GP or investigating physician if there are no other reasonable causes explaining these deviations.

The association of non-specific symptoms and biochemistry factors with cancer diagnosis may vary between patients with and without comorbidities, as some of these comorbidities might explain some symptoms and abnormal biochemistry levels. This is however not within the scope of this study but would be addressed in future prospective studies.

According to national guidelines cytopenia in two or three cell-lines is regarded as criteria for referral to a haematological CPP [45–48]. Results of the sensitivity analysis showed that anemia, leukocytopenia or thrombocytopenia were suggestive of a haematological cancer diagnosis, supporting the guidelines of referral to the haematological CPP.

### Strengths and limitations

The retrospective design in a clinical set-up with physician driven decisions caused high numbers of missing values in objective examinations. This could have caused an overestimation of effects, and was handled by not including objective findings in the analysis of association with cancer and by simply describing the use of and findings by imaging. Comorbidities could also have been insufficiently reported in the patient files leading to an underestimation of the effect of comorbidities. Reportings of comorbidities were however unlikely to have been unevenly distributed in the two groups. Information registered in the patient files might have been misinterpreted, as the information was not collected with the sole purpose of this study.

Due to the retrospective design it was difficult in this study to assess which patients were most likely to develop a cancer diagnosis. In order to identify high risk and low risk patients in this group with otherwise non-specific symptoms, prospective studies are needed - preferably multicentre studies including collaboration with GPs in order to assess the prevalence of risk factors and predict the risk of cancer.

A major strength was the population size with 825 included patients. Patients were unselected thus describing the everyday clinical situation, including patients referred both from the GP, specialist medical practitioners and hospital departments. Broad inclusions make the results of this study generalizable to the clinical practice at the NSSC-CPP and in part to the referring units.

Patients were followed for an entire year from time of cancer diagnosis, giving an excellent follow-up and making it possible to estimate one-year mortality. Diagnoses were crosschecked both in paper files, electronic patient files and in the patient index, rather than relying on registers accuracy, thus ensuring a strong link between the investigational course and the diagnosis found to be the most likely cause of symptoms. The collection of data furthermore led to another study exploring quality of life in patients referred to NSSC-CPP [49].

## Conclusion

The prevalence of cancer in patients seen at the NSSC-CPP is substantial. Non-specific symptoms should raise awareness in the general population and by the GP even though no single symptom was associated with cancer. Anemia, leukocytosis, thrombocytopenia and elevated LDH and CRP should raise clinical concern in patients with non-specific symptoms where the suspicion of cancer has been raised, and could possibly guide the physician towards the most likely diagnosis and the best-suited investigational course. An alarmingly high one-year mortality of 44% in this population suggests that cancer diagnoses were found at late stages. Larger and prospective studies are needed to identify combinations of symptoms, findings and biochemistry related to a cancer diagnoses, hopefully making it possible to find cancer diagnoses in these patients at earlier stages.

## Abbreviations

2WW: 2-week wait
CI: 95% Confidence Interval
CPP: Cancer patient pathway
CRP: C-reactive protein
CT: Computed tomography
FDG-PET/CT: 18F–fluorodeoxyglucose positron emission tomography in combination with CT
FUO: Fever of unknown origin
GP: General practitioner
ICD10: International classification of Diseases 10th revision
IQR: Inter Quartile Range
LDH: Lactate dehydrogenase
MRI: Magnetic resonance imaging
NOH: North Zealand Hospital, Hillerød, Denmark
NSSC-CPP: Cancer patient pathway for patients with non-specific symptoms and signs of cancer
SOP: Standard Operating Procedure
UK: United Kingdom
